# An assessment of the current epidemiological and laboratory capacities for influenza‐like illnesses and severe acute respiratory infection surveillance, Yemen 2022

**DOI:** 10.1111/irv.13130

**Published:** 2023-04-13

**Authors:** Ali A. Al‐Waleedi, Ahmed A. Thabet, Nasreen bin Azoon, Adham Dandarwe, Abed Salem Al‐Amoudi, Amar Al‐Gailani, Bakil Atef

**Affiliations:** ^1^ Aden University 00967 Aden Yemen; ^2^ WHO 00967 Aden Yemen; ^3^ Department of Disease Control and Surveillance Influenza Program Focal Point, MOPH&P 00967 Aden Yemen; ^4^ Department of Disease Control and Surveillance MOPH &P 00967 Aden Yemen

**Keywords:** assessment, influenza surveillance, SARI/ILI, sentinel site, Yemen

## Abstract

**Background and Objectives:**

We aim to re‐activate influenza sentinel surveillance system in Yemen after disruption related to repurposing for COVID‐19 pandemic. WHO Country Office (CO) in collaboration with Yemen's Ministry of Public Health and Population (MOPH&P) jointly conducted an assessment mission to assess the current situation of the influenza sentinel surveillance system and assess its capacity to detect influenza epidemics and monitor trends in circulating influenza and other respiratory viruses of epidemic and pandemic potential. This study presents the results of the assessment for three sentinel sites located in Aden, Taiz, and Hadramout/Mukalla.

**Methodology:**

A mixed methods approach was used to guide the assessment process and to help achieve the objectives. Data were collected as follows: desk review of the sentinel sites records and data; interviews with stakeholders, including key informants and partners; and direct observation through field visits to the sentinel sites, MOPH&P and the Central Public Health Laboratory (CPHL). Two assessment checklists were used: assessment of sentinel sites for SARI surveillance, and checklist for assessment of availability of SARI sentinel surveillance.

**Results and Conclusion:**

COVID‐19 has affected health systems and services, and this was demonstrated in this assessment. The influenza sentinel surveillance system in Yemen is not effectively functional; however, there is plenty of room for improvement if investment in the system's restructuring, training, building technical and laboratory capacities, and conducting continuous and regular supervision visits.

## 1 INTRODUCTION

The conflict in Yemen entered its eighth year with the resulting humanitarian crisis in terms of food security, people in need, and displacements.^1^ The health system has collapsed with serious shortages of medicines and medical supplies and shattered essential services that are unable to accommodate, provide or respond to the needs of the populations.^2^, ^3^, ^4^, ^5^, ^6^ Furthermore, the COVID‐19 pandemic has added an extra burden to health systems in Yemen and globally. The pandemic has highlighted the need to maintain essential public health functions and services. It has also highlighted the critical role of public health professionals in combating epidemics and pandemics.^7^, ^8^

Influenza surveillance in Yemen was initially introduced in 2010 following the H1N1 outbreak by setting up two sentinel sites for Severe Acute Respiratory Infection (SARI) in collaboration with NAMRU‐3^9^ and World health Organization (WHO). In 2014 and 2021, the influenza sentinel surveillance system expanded to include another three sentinel sites in Taiz, Hodeida, and Hadramout‐Mukalla following the support that from the Pandemic Influenza Preparedness (PIP) Framework Partnership Contribution Funds.^2^

The objective of the SARI/influenza‐like illnesses (ILI) surveillance system is the following: strengthen the influenza surveillance mechanisms in the country in order to provide forewarning of outbreaks and emergence of novel influenza virus; expand the laboratory surveillance of influenza for confirmation of ILI and use it to estimate the proportion of these cases that are due to influenza; provide viral isolation to the WHO Influenza Reference Laboratory for antigenic analysis to detect new strains and for vaccine formulation; and, firm up national vaccine policy based on the local circulating influenza virus.^3^

In order to re‐activate the influenza surveillance system after repurposing it for the COVID‐19 pandemic, to improve influenza case detection and monitoring of influenza epidemics, and to monitor trends in circulating influenza viruses, the WHO in collaboration with Yemen's Ministry of Public Health and Population (MOPH&P) led a mission to conduct an assessment of the current situation of epidemiological and laboratory surveillance in Yemen. The focus of this mission was related to the influenza surveillance system's capacity to detect and monitor influenza epidemics and to monitor trends in circulating influenza viruses. The following work presents the results of the assessment for three sentinel sites located in Aden, Taiz, and Hadramout/Mukalla.

## 2 METHODOLOGY

### 2.1 Design

A mixed methods approach was used to guide the assessment process and to help achieve study objectives. Data were collected from the following sources: desk review of the sentinel sites records and data; interviews with key stakeholders, including informants and partners; direct observation through field visits to the sentinel sites, MOPH&P and the Central Public Health Laboratory (CPHL).

### 2.2 Data collection

Two assessment checklists were used for the purpose of this assessment: ‘Assessment of sentinel sites for SARI surveillance’ (see Appendix A) and checklist for ‘Assessment of availability of SARI sentinel surveillance’ (see Appendix B).

The ‘Assessment of sentinel sites for SARI Surveillance’ checklist was used to collect information during meetings and discussions with key informants from relevant staff in the MOPH&P and CPHL. This was supplemented by supervisory visits to the sentinel sites and laboratories in Aden (Al‐Sadaka Teaching Hospital), Taiz (Al‐Jamhori Hospital), and Hadramout/Mukalla (Ibin Sayna Teaching Hospital) in August and September 2022. The process of data collection commenced in August 2022 and was completed in September by an expert team from the Yemen WHO‐CO, and the Ministry of MOPH&P led the data collection.

### 2.3 Ethical considerations

Approval to conduct the study was given by the Ministry of Public Health and Population in Yemen‐Aden.

## 3 RESULTS

At present, sentinel data are collected from inpatient departments at the sentinel sites. The information collected is minimal and includes number of consultation of SARI cases by age group and clinical/laboratory data, which include the following: date of onset, date of specimen collection, and date of testing. All sentinel sites are committed to send their SARI report to the CPHL and MOPH&P on a weekly basis. Selected sites are both SARI and ILI sites but currently ILI enrolment of cases is not functional yet and so there are no specimens collected from outpatient departments.

**FIGURE 1:**
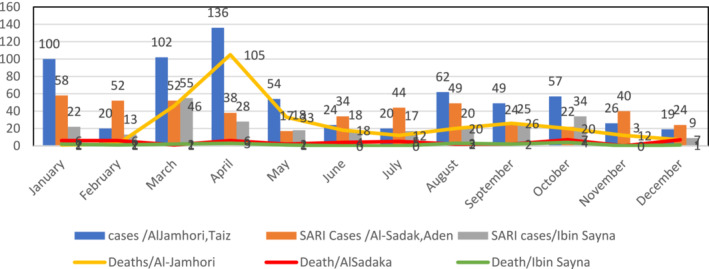
Distribution of SARI cases and deaths in selected sentinel sites, Yemen 2021.

Every day, a dedicated form of electronic integrated surveillance system line list for SARI is filled in the sentinel sites, which adheres to the WHO–SARI case definition. Data are sent on a weekly basis to the MOPH&P health staff at central level. Figure 1 illustrates the inpatients meeting the SARI case definition cases and deaths in the targeted three sentinel sites: Aden, Taiz, and Hadramout/Mukalla for 2021.

Epidemiological data demonstrated that SARI incidence is spread out over the country and not just the central region. During the COVID‐19 pandemic, WHO supported National Public Health Laboratories in Yemen with PCR unit, and thus, all designated sentinel sites for SARI are equipped with PCR machines; staff can test influenza samples depending on reagent availability. In addition, two central public health laboratories in Aden and Sanaa are supplied with nano sequencers and designated as a referral laboratory. CPHL in Yemen is not yet recognized as a National Influenza Center (NIC). In Sana'a, virus isolation capacity exists as well, but it is not currently functioning. Sequencing capacity exists in Aden and Sana'a since September 2022.

Veterinary Services Department (DVS) in the Ministry of Agriculture is also actively monitoring the influenza activity in poultry rearing for the livestock market in the country. However, there is no network established between MOPH&P and DVS, which is usually only enhanced and activated when a zoonotic infection takes place globally or locally. In terms of private clinics and hospitals, there is no current system established to collect any data for SARI/ILI to merge with the government's statistics. This is a growing issue as the users of the private sector are usually those who can afford to pay more and are more likely to make trips overseas—as such, data compiled at the central level do not represent an accurate picture of the whole population.

### 3.1 National, provincial, and field level(s) influenza surveillance system in Yemen

Information communication between various administrative levels is a great drawback; the flow of information from the field to provinces is inconsistent at best. This incompleteness of data is due to inadequate communication and results in poor data analysis, which often does not transpire in the first place, and naturally there are no results to be shared back to provinces and clinics. This generates frustration among the health personal and a sentiment of lack‐of‐purpose for data collection.

The division of tasks between field, district, and provincial teams is not always clear. In some instances, clinic‐based personnel are the ones compiling daily data in weekly reports, whereas in other cases, it is the district monitoring and evaluation personnel that takes on this task. The same issues were observed between district and provincial teams; provincial teams are meant to send weekly compilations to the national team; however, they sometimes do it only monthly and sometimes not at all. At the national level, the personnel in charge of collecting and analyzing data were unable to demonstrate weekly/monthly/yearly compilations; they were also unable to share calculations of percentages for indicators for each site. Reporting rates were low and data quality was questionable.

The assessment of sentinel sites in Aden (Al‐Sadaka Teaching Hospital), Taiz (Al‐Jamhori Hospital), and Hadramout/Mukalla (Ibin Sayna Teaching Hospital) showed that the hospital management was agreeable in terms of implementing SARI/ILI surveillance and that the hospital staff were willing to work with influenza surveillance. All hospitals offer outpatient and inpatient services for those in the catchment area, and from all over Yemen. They employ permanent trained clinical and laboratory staff for SARI/ILI surveillance. All hospitals are teaching and referral ones.

Unlike Ibin Sayna Hospital (Mukalla), Al‐Jamhori hospital (Taiz) and Al‐Sadaka hospital (Aden) have significantly better data quality. This is due to the availability of well‐trained staff in charge of filling in the SARI forms. Completeness of records in Aden made it feasible for the SARI logbook details for inpatient consultation to be available; data for each calendar year and reporting data for SARI (age, gender, date of onset of symptoms, symptoms and signs, patient temperature, and final diagnosis) were complete. This is unfortunately not the case in Taiz and Mukalla, whereby health personnel find the SARI/ILI data collection to represent a significant added workload especially due to the lack of electronic records or an internet connection; this makes their data recording and registration laborious and time consuming.

The quality of the data collected is quite poor due to incompleteness, and the logbook for SARI data per calendar year is either unavailable or incomplete. Laboratory equipment and infrastructure also differ between both sites; the laboratory in Aden is well equipped with RT‐PCR 7500 and refrigerators for sample storage at 4°C and −70°C, though it sometimes runs out of reagents. On the contrary, in Taiz and Mukalla, the hospital does not have a fridge where the sample specimens can be kept, and the laboratory has no capacity to test for influenza by PCR.

The current assessment of the SARI sentinel sites also showed that the feasibility and sustainability of the chosen facilities are sub‐optimal. This is due to a variety of factors: first and foremost, the lack and incompleteness of efficient, consistent, and sustainable mechanisms for collection, storage, and transport of clinical specimens. This is compounded by the lack of stable and long‐term funding to cover hazard pays or incentives for well trained staff, in addition to the general cost of the surveillance operations at the sites and the incapability of the existing infrastructure to cover and facilitate the necessary mechanisms for data reporting.

## 4 DISCUSSION

COVID‐19 has affected health systems and services, and this was demonstrated from the findings of this assessment. The influenza sentinel surveillance system in Yemen is not effectively functional. However, there's plenty of room for improvement if there is investment in the system's restructuring, training, building technical and laboratory capacities, and conducting continuous and regular supervision visits.

Yemen has currently reactivated only three sentinel sites in the southern and eastern part for SARI, and the system does not include private clinics or hospitals. There is an urgent need to collect SARI/ILI data from more sentinel sites including private clinics and hospitals to represent the entire population. There is also a need to have a National Coordinating Influenza Laboratory (NCIL), which will coordinate the clinical specimens from all governmental laboratories involved in laboratory‐based influenza virus surveillance in the country and to coordinate all the laboratory activities pertaining to influenza including training and coordinating sending of specimens to WHO Collaborating Centers for influenza for sub‐typing & identification of new strains of influenza viruses.

Furthermore, it is worth mentioning that monitoring the evolution of the pandemic, influenza preparedness (PIP) situation in Yemen should consider the following for each influenza pandemic periods & phases: the type of surveillance system required, the direction in which a change is expected, and the frequency and intensity of variations to be expected. Yemen has four seasons and influenza virus is most likely circulating all year round, therefore there is greater probability of getting different patterns of circulating influenza virus around the year. As such, and given the successful collaboration between MOPH&P in Yemen with other sectors during the COVID‐19 pandemic, the surveillance department at the Ministry proposed to include influenza in the Joint Program Review Mission (JPRM).

### 4.1 Limitations

Due to the security situation and war, the authors would like to caution that the assessment did not include more sites (public and private) from different geographical locations, so the representativeness is limited and cannot be generalized for all Yemeni population. Furthermore, the researchers found that the lack of SARI reagents in the mentioned and assisted sentinel site resulted in constraints to properly assess staff performance and quality of equipment (for example PCR). The researchers also encountered hurdles in relation to the lack of transparency and accountability, which limits SARI data quality.

### 4.2 Recommendations

In accordance with the insight provided from this assessment in terms of the current situation of epidemiological and laboratory surveillance related to capacity to detect and monitor influenza epidemics and to monitor trends in circulating influenza viruses, a variety of action points for further development are put forward. Primarily, a great deal of investment in training and capacity building is absolutely crucial; trainings workshops can be conducted for surveillance/laboratory staff on SARI/ILI data management and reporting—this should be coupled with training of laboratory personnel to build their capacity to use PCR to test for influenza and to improve the capacity of identified laboratories and surveillance sites, in terms of infrastructure, human and financial resources in order to expand and strengthen the surveillance of influenza. This will not be possible without stable and long‐term funding to cover the general cost of the surveillance operations and training.

On a coordination front, expansion of the clinical and laboratory SARI/ILI sentinel sites to include the private health sector in order to serve all population in Yemen is important. This will result in a more comprehensive information, which is representative of the general populations' disease trends. Such coordination should also be apparent at the central level, where a network between MOH (Disease Surveillance and Control) and Ministry of Agriculture (Department of Veterinary Disease Surveillance) is also necessary. This can be facilitated by the introduction of an electronic registry system in the sentinel sites to enable more efficient data sharing and communication between all the stakeholders. As mentioned earlier, there is a need to establish a National Coordinating Influenza Laboratory (NCIL), which will coordinate the clinical specimens from all governmental laboratories involved in laboratory‐based influenza virus surveillance in the country and to coordinate all the laboratory activities pertaining to influenza.

Finally, and to ensure smooth operational flow, clear and transparent task allocation between field, district, and provincial ILI/SARI surveillance is necessary. This will enable efficient, consistent, and sustainable mechanisms for collection, storage, and transport of clinical specimens, as well as completeness of data registers.

## AUTHOR CONTRIBUTIONS

**Ali A. Al‐Waleedi:** review and edited all stages of the research and obtained official approval. **Ahmed A. Thabet:** managed all stages of research from proposal conceptualization to study design, trained data collectors, supervised fieldwork, formal analysis and data interpretation and wrote first and final drafts of report. **Nasreen bin Azoon:** prepared questionnaires, trained data collectors, reviewed methodology and data analysis, supervised field work and reviewed final report. **Adham Dandarwe:** reviewed and edited all stages of the research. **Abed Salem Al‐Amoudi:** data collector and analysis. **Amar Al‐Gailani:** formal data analysis and developed questionnaire. **Bakil Atef:** collected data and reviewed data analysis. All authors give approval of the final version to be published.

## CONFLICT OF INTEREST STATEMENT

The authors declare that they have no competing interests.

### PEER REVIEW

The peer review history for this article is available at https://www.webofscience.com/api/gateway/wos/peer-review/10.1111/irv.13130.

## Data Availability

We certify that all data are available and kept in MOPH influenza program. The data could be share under request for any review or audit.

## Appendix A ASSESSMENT OF SENTINEL SITES FOR SARI SURVEILLANCE

Country: _____________ Province/Governorate: _______________ District: _____________

Health Facility name: ___________________________.

Type of the health facility: General or Specialized hospital: _____________________.

What are the other main hospitals in the area? __________________________________________.

Name of selected person for correspondents: __________________________________.

E‐mail address: ________________________________ Phone No: ______________________

Assessor's name: _____________________ Organization: ____________ E‐mail address: _________________

Date of assessment (dd/mm/yy): //.

- Site description:

1. Is the hospital management agreed to implementing Influenza Surveillance? Yes No.
2. Does the site offer outpatient services? Yes NO.
3. Does the health facility have logbook to maintain data at outpatient, inpatient wards and lab? Yes No.
4. Refer to Q3, if the logbooks partially available in some sites, please write them … … ………………………………………………………………… ….
5. Refer to Q3, check if it includes age, gender, date of onset of symptoms, symptoms and signs, patient temp, professional and final diagnose) … … ………………………………………………………………………………………………………………………………………………………………………………………… … …
6. What is the monthly outpatient consultation cases and deaths for the last year (please fill the below table)?
  Year ___.
  **Table:**
  Jan		Feb		Mar		Apr		May		Jun	
  Cases	Deaths	Cases	Deaths	Cases	Deaths	Cases	Deaths	Cases	Deaths	Cases	Deaths
  Jul		Aug		Sep		Oct		Nov		Dec	
  Cases	Deaths	Cases	Deaths	Cases	Deaths	Cases	Deaths	Cases	Deaths	Cases	Deaths
7. Does the site offer inpatient services? Yes No
8. What is the monthly inpatient consultation cases and deaths for the last year (please fill the below table)?
  Year _________________.
  **Table:**
  Jan		Feb		Mar		Apr		May		Jun	
  Cases	Deaths	Cases	Deaths	Cases	Deaths	Cases	Deaths	Cases	Deaths	Cases	Deaths
  Jul		Aug		Sep		Oct		Nov		Dec	
  Cases	Deaths	Cases	Deaths	Cases	Deaths	Cases	Deaths	Cases	Deaths	Cases	Deaths
9. Are patients of <5 years and >5 year (all age group) attending the hospital? Yes No
10. Are the patients attending the hospital including pregnant mothers? Yes No
11. Are patients from all ethnic groups and socioeconomic strata attending the clinic? Yes No
12. Is the health facility part of the national surveillance system? Yes No
13. Is it possible to estimate the catchment population area of this heath facility? Yes No
14. If yes: what is the catchment population number of the area? … … ………………… …
15. Are patients who fit with SARI case definition admitted in this health facility? Yes No
16. If yes, what is the monthly inpatient consultation of those who met SARI case definition
  *
  SARI case definition: patients who has acute respiratory illness + fever = >38C + cough + onset with the last 10 days + required admission.
   cases and deaths for the last year (please fill the below table)?
  Year _________________.
  **Table:**
  Jan		Feb		Mar		Apr		May		Jun	
  Cases	Deaths	Cases	Deaths	Cases	Deaths	Cases	Deaths	Cases	Deaths	Cases	Deaths
  Jul		Aug		Sep		Oct		Nov		Dec	
  Cases	Deaths	Cases	Deaths	Cases	Deaths	Cases	Deaths	Cases	Deaths	Cases	Deaths
17. Is any data analysis performed at the health facility level? Yes No
18. Please check the inpatient logbook, verify are the data entry of the patients entered timely (select 5 from the current inpatient and check if their data has been included to the logbook)
19. How many beds for admission? ___________________________
20. How many beds in the ICU (Intensive care Unit)? ________________________
21. How many cases and deaths of pneumonia admitted in the ICU last year (fill below table)

Year _________________.

**Table:**

Jan		Feb		Mar		Apr		May		Jun	
Cases	Deaths	Cases	Deaths	Cases	Deaths	Cases	Deaths	Cases	Deaths	Cases	Deaths
											
Jul		Aug		Sep		Oct		Nov		Dec	
Cases	Deaths	Cases	Deaths	Cases	Deaths	Cases	Deaths	Cases	Deaths	Cases	Deaths
											

- Human resources capacity:

1. Does the site have at least one permanent medical staff in the identification of ILI and SARI and in respiratory sample collection? Yes no
2. Does the health facility have at least one surveillance officer/statistician who has been trained on data collection, entering and sending the data to the upper level (district surveillance officer or national surveillance officer)? Yes No
3. Does the health facility have at least one permanent lab worker who can be trained in the collection, storage, testing, and transportation of respiratory sample collections? Yes No

- Infrastructure

1. Does the surveillance staff have access to internet? Yes No
2. Does the site have a reliable power supply and fridge where the sample specimens can be kept? Yes No
3. Does the site have a laboratory? Yes No
4. Does the lab have the capacity to test for influenza by PCR? Yes No
5. If the answer of Q4 is yes, what are the results of last year tested cases that fit the SARI case definition?

**Table:**

Influenza A	Influenza A (H1N1)pdm09	Influenza A (H3N20	Influenza B	Other type specify	
				Type	No of pos.
					
					

1. Does the health facility have refrigerator for sample storage at 4°C? Yes No
2. Does the health facility have fridge (−20°C)? Yes No

## Appendix B CHECKLIST FOR SELECTING SENTINEL SITES FOR INFLUENZA (SARI/ILI)

**Table:**

Site description	
Is hospital management agreeable to implementing influenza surveillance?	Yes () NO ()
Is the staff willing to work with influenza surveillance?	Yes () NO ()
Does the site offer outpatient services?	Yes () NO ()
Does the site offer inpatient services?	Yes () NO ()
Are patients attending the clinic are from all age groups?	Yes () NO ()
Are patients attending from all socio‐economic strata and ethnic groups?	Yes () NO ()
What is the 3‐month average number of outpatient consultations?	
What is the 3‐month average number of in‐patient medical admissions?	
Can the catchment population of the site be estimated?	Yes () NO ()
Human Resource Capacity	
Does the site have at least permanent clinical staff that could be trained in the identification of ILI and SARI and respiratory sample collection?	Yes () NO ()
Does the site have at least one permanent lab worker that can be trained in the collection, storage, testing and transportation of respiratory sample specimens?	Yes () NO ()
Infrastructure	
Does the site have laboratory?	Yes () NO ()
Does the surveillance staff have access to computers?	Yes () NO ()
Does the surveillance staff have access to the internet?	Yes () NO ()
Does the site have a reliable power and fridge where the sample specimens can be kept?	Yes () NO ()
